# Chest tube placement in trauma patients: please use sonography

**DOI:** 10.1186/s12245-024-00596-3

**Published:** 2024-02-13

**Authors:** Fatimaezzahra Saroukh, Ayoub Bouchama, Ayoub Belhadj, Younes Aissaoui

**Affiliations:** 1 Department of Intensive Care Medicine, Avicenna Military Hospital, Marrakesh, Morocco; 2 Biosciences and Health Research Unit, Faculty of Medicine and Pharmacy Marrakech, Cadi Ayyad University, Marrakesh, 40000 Morocco

## Case presentation

**Fig. 1:**
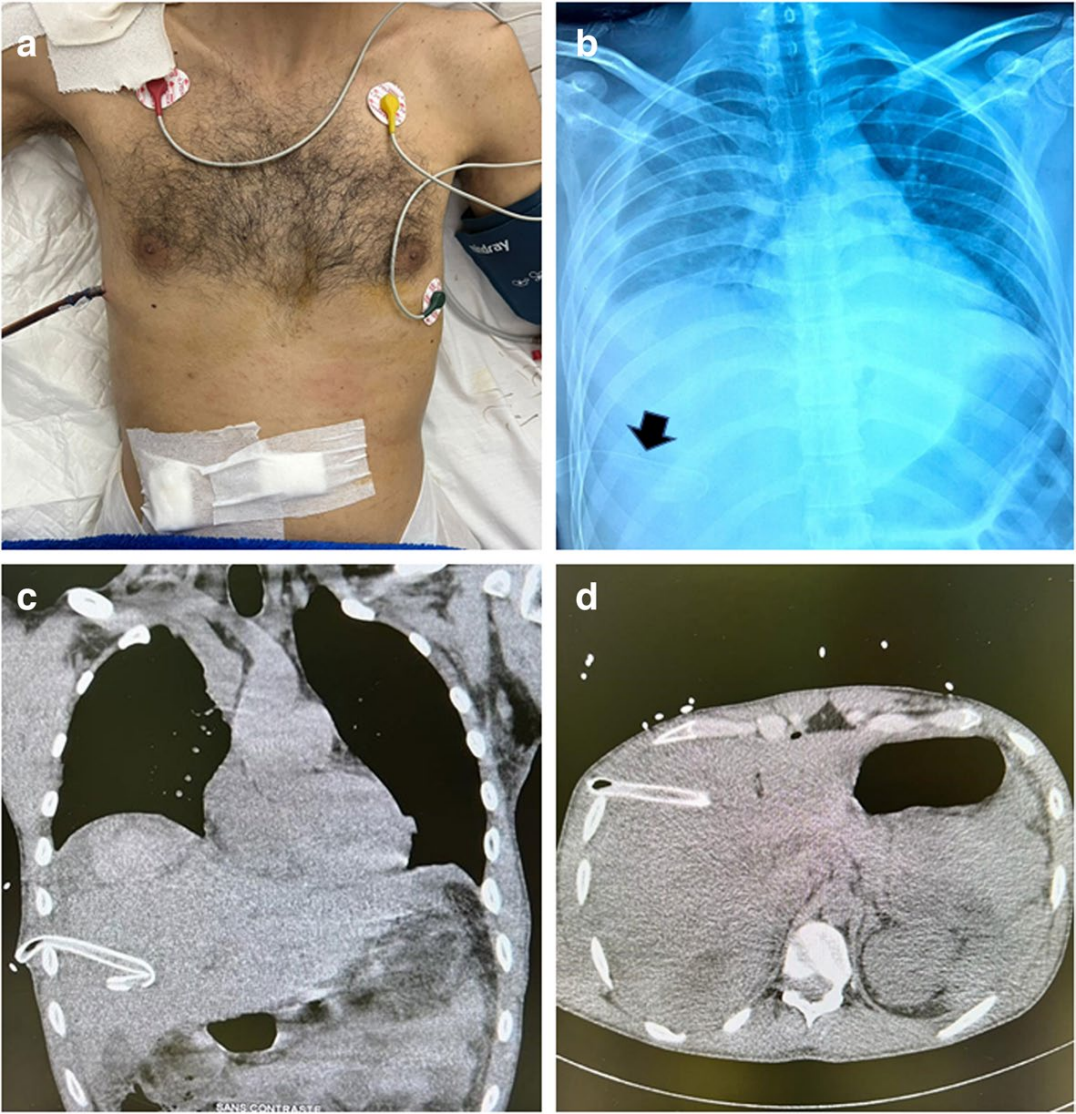
Illustrations of the insertion site (panel A), chest X-ray (panel B), and abdominal CT scan (panels C and D) depicting an intrahepatic aberrant chest tube highlighted by a black arrow in the chest X-ray

A 27-year-old patient with no known medical history was admitted for polytrauma resulting from a traffic accident. The initial thoraco-abdominal computed tomography (CT) scan revealed a minimal left pneumothorax, liver lacerations with intracapsular hematoma, and moderate hemoperitoneum. After 48 h, the patient’s respiratory status worsened. A subsequent chest CT scan identified a medium-sized right hemothorax leading to the insertion of a 28-Fr chest tube (Fig. 1, panel A). Approximately 400 mL of blood flowed freely through the chest tube before ceasing spontaneously. However, the chest tube was found to be dangerously low in its placement.

## Diagnosis

A follow-up chest X-ray confirmed the misplaced chest tube, located intraabdominally (Fig. 1, panel B). Subsequent abdominal CT imaging confirmed the tube’s intrahepatic placement (Fig. 1, panels C and D). The blood flow through the intrahepatic tube likely corresponded to the intracapsular hematoma. The malpositioned tube was carefully removed under strict monitoring, and another chest tube was inserted under ultrasound (US) guidance, with the surgical team prepared for intervention if needed. The patient’s recovery was uneventful, and he was discharged after a few days.

**Fig. 2:**
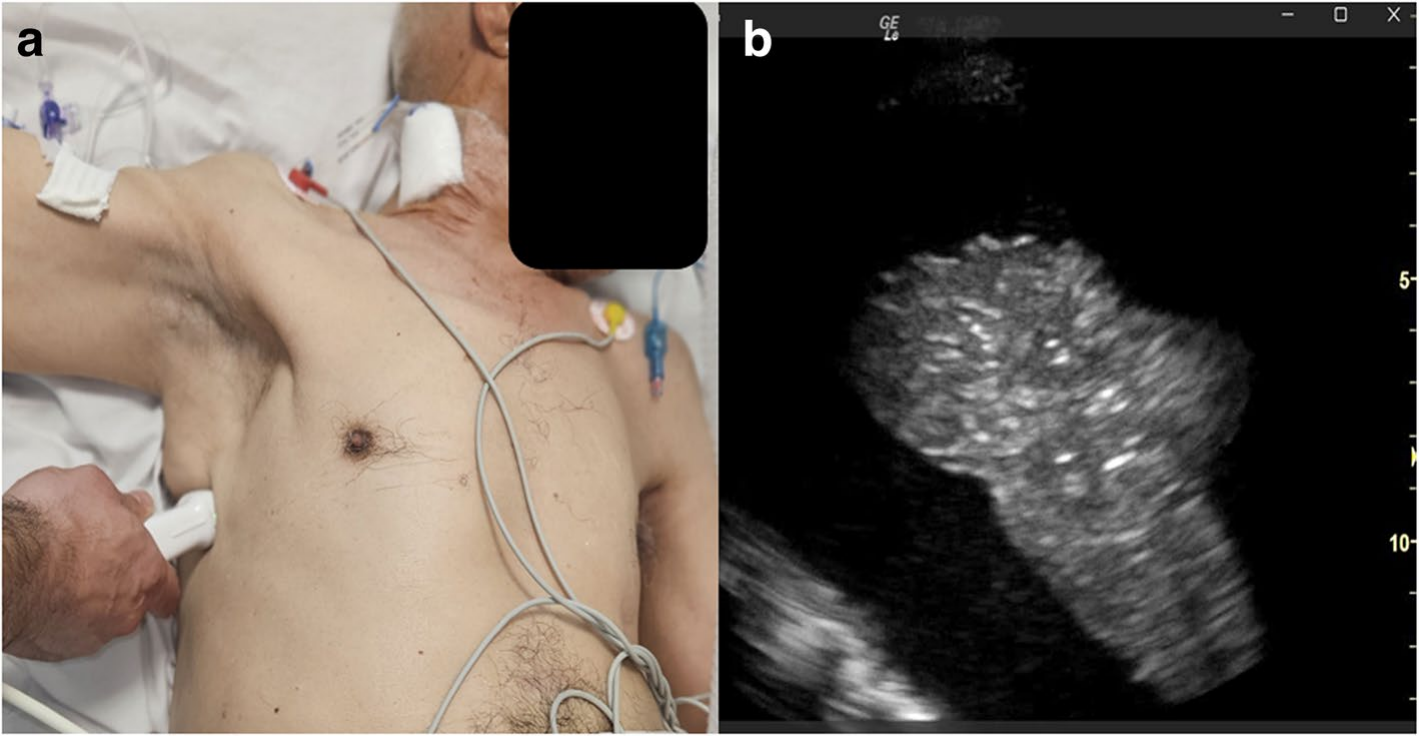
1 Illustration depicting the recommended probe position during chest ultrasonography for pleural effusion drainage. Either a phased array or curved probe can be utilized; in this instance, a phased array probe was employed. 2 Chest ultrasound (intercostal view) displaying a substantial pleural effusion alongside a consolidated lung

This case underscores the value of using US guidance to prevent chest tube misplacement and emphasizes that relying solely on anatomical landmarks can lead to errors. In fact, ultrasound guidance allows the choice of the best puncture site and provides real-time visualization of the chest structures (Fig. 2, panels 1 and 2) [1]. Ultrasound guidance for chest drainage should be incorporated into routine medical practice [2]. However, comparative studies are needed to determine whether these approaches should be established as standard care.

This case highlights the importance of using US guidance to prevent chest tube misplacement, emphasizing that reliance solely on anatomical landmarks can lead to errors. Ultrasound guidance enables the selection of the best puncture site and provides real-time visualization of chest structures (Fig. 2, panels 1 and 2) [1]. Incorporating ultrasound guidance for chest drainage into routine medical practice is recommended. However, further comparative studies are necessary to determine whether these approaches should be established as standard care [2].

## Data Availability

Data sharing is not applicable to this article as no datasets were generated or analyzed during the current study.
